# COVID-19 Disease Severity and Death in Relation to Vitamin D Status among SARS-CoV-2-Positive UAE Residents

**DOI:** 10.3390/nu13051714

**Published:** 2021-05-19

**Authors:** Habiba AlSafar, William B. Grant, Rafiq Hijazi, Maimunah Uddin, Nawal Alkaabi, Guan Tay, Bassam Mahboub, Fatme Al Anouti

**Affiliations:** 1 Center for Biotechnology, Khalifa University of Science and Technology, Abu Dhabi 127788, United Arab Emirates; habiba.alsafar@ku.ac.ae (H.A.); guan.tay@uwa.edu.au (G.T.); 2 Department of Biomedical Engineering, College of Engineering, Khalifa University of Science and Technology, Abu Dhabi 127788, United Arab Emirates; 3 Department of Genetics and Molecular Biology, College of Medicine and Health Sciences, Khalifa University of Science and Technology, Abu Dhabi 127788, United Arab Emirates; 4 Sunlight, Nutrition and Health Research Center, P.O. Box 641603, San Francisco, CA 94164-1603, USA; williamgrant08@comcast.net; 5 Department of Mathematics and Statistics, College of Natural and Health Sciences, Zayed University, Abu Dhabi 144534, United Arab Emirates; Rafiq.Hijazi@zu.ac.ae; 6 Department of Pediatric Infectious Disease, Sheikh Khalifa Medical City, Abu Dhabi 51900, United Arab Emirates; muddin@seha.ae (M.U.); nalkaabi@seha.ae (N.A.); 7 Division of Psychiatry, Faculty of Health and Medical Sciences, University of Western Australia, Crawley, Western Australia, Australia; 8 School of Medical and Health Sciences, Edith Cowan University, Joondalup, Western Australia, Australia; 9 Dubai Health Authority, Rashid Hospital, Dubai, United Arab Emirates; drbassam_mahboub@yahoo.com; 10 Department of Health Sciences, College of Natural and Health Sciences, Zayed University, Abu Dhabi 144534, United Arab Emirates

**Keywords:** vitamin D, COVID-19, SARS-CoV-2, severity, mortality, United Arab Emirates

## Abstract

Insufficient blood levels of the neurohormone vitamin D are associated with increased risk of COVID-19 severity and mortality. Despite the global rollout of vaccinations and promising preliminary results, the focus remains on additional preventive measures to manage COVID-19. Results conflict on vitamin D’s plausible role in preventing and treating COVID-19. We examined the relation between vitamin D status and COVID-19 severity and mortality among the multiethnic population of the United Arab Emirates. Our observational study used data for 522 participants who tested positive for SARS-CoV-2 at one of the main hospitals in Abu Dhabi and Dubai. Only 464 of those patients were included for data analysis. Demographic and clinical data were retrospectively analyzed. Serum samples immediately drawn at the first hospital visit were used to measure serum 25-hydroxyvitamin D [25(OH)D] concentrations through automated electrochemiluminescence. Levels < 12 ng/mL were significantly associated with higher risk of severe COVID-19 infection and of death. Age was the only other independent risk factor, whereas comorbidities and smoking did not contribute to the outcomes upon adjustment. Sex of patients was not an important predictor for severity or death. Our study is the first conducted in the UAE to measure 25(OH)D levels in SARS-CoV-2-positive patients and confirm the association of levels < 12 ng/mL with COVID-19 severity and mortality.

## 1. Introduction

COVID-19 is a complex respiratory syndrome caused by the severe acute respiratory syndrome coronavirus 2 (SARS-CoV-2), an enveloped RNA virus extremely transmissible through respiratory aerosols [1]. This virus, which can lead to pulmonary failure and fatality, has a noticeable genetic similarity to the beta-coronaviruses that cause SARS and Middle East respiratory syndrome [2]. The clinical indications of COVID-19 disease range from asymptomatic to mild to severe. Although most affected patients develop mild symptoms, about 5% of cases might progress to acute respiratory distress syndrome, requiring hospitalization and intensive care [3]. Elevated oxidative stress levels, exaggerated immune response due to the cytokine storm, and uncontrollable liberation of proinflammatory cytokines, along with the activation of pre-coagulating factors, all contribute to severe inflammation, which is exaggerated in acute respiratory distress syndrome [4,5]. Vitamin D is a fat-soluble prohormone steroid that has endocrine, paracrine, and autocrine functions [6]. Recent studies demonstrated that vitamin D could mediate antiviral activity by many actions, including enhancing apoptosis and autophagy as well as by inducing antimicrobial peptides [7,8].

Accumulating evidence has shown that severe disease is high among vulnerable populations: the elderly and patients with chronic diseases such as asthma, cancer, chronic obstructive pulmonary disease, diabetes, and hypertension. People who are obese or belong to ethnic groups with darker skin also experience more severe disease [9,10].

To fully decipher the mechanism underlying COVID-19 disease susceptibility, researchers are considering several possible contributing factors [11]. Vitamin D deficiency has emerged as a leading candidate [7,12,13]. Although concrete evidence about vitamin D’s therapeutic role in COVID-19 has yet to be confirmed through randomized controlled trials (RCTs), vitamin D is associated with protective effects [7]. Such effects arise because vitamin D, as an essential prohormone that maintains bone homeostasis, also mediates many important non-skeletal functions, including modulating the immune system [14]. 

Several studies have documented the correlation between vitamin D deficiency and severity of viral infections such as influenza [15]. A study among children and adolescents indicated a higher risk of viral respiratory tract infections with deficient and insufficient serum 25-hydroxyvitamin D [25(OH)D] levels [16]. Moreover, a meta-analysis by Martineau and colleagues of RCTs across the globe including 11,321 participants showed that vitamin D-deficient patients had better protection against respiratory tract infections after supplementation with vitamin D (odds ratio (OR) = 0.30; 95% confidence interval (CI), 0.17, 0.53) [17]. Recently, vitamin D was identified by genomics-guided tracing research to be involved in regulating gene expression with potential to alleviate SARS-CoV-2 infection upon binding to the vitamin D response element [18]. The well-established role of vitamin D as an anti-inflammatory agent explains the beneficial effect of vitamin D in both the innate and adaptive immune responses and in producing antimicrobial agents cathelicidin (LL-37) and human β-defensin 2 [19,20].

Moreover, vitamin D regulates the renin–angiotensin system and expression of angiotensin-converting enzyme 2 (ACE2), and the corresponding cell receptor, which mediates coronavirus infection (ACE2 and the ACE2 receptor are distinct, and ACE2 seems able to bind SARS-CoV-2, preventing it from attaching to the ACE2 receptor). Elevated expression of ACE2 had been linked to a protective effect in the lungs during acute injury. Higher expression also reduces infectivity of SARS-CoV-2 by attenuating attachment to ACE2 receptors in target cells [21,22].

A previous study that examined the expression pattern for ACE2 in a mouse model in the context of aging and sex showed a significantly downregulated expression for ACE2 in older female and male rats, by 67% and 78%, respectively [23]. That decrease of ACE2 protein accords with the reported higher risk of COVID-19 infection and severity of disease among males [22]. Vitamin D also strengthens the epithelial physical barrier through its effect on E-cadherin, which tightens the cellular junctions to be tight and effective in impeding viral particles from penetrating the lungs [20]. Evidence from studies in 20 European countries showed that 25(OH)D concentrations and COVID-19 mortality were inversely associated, as well as that vitamin D deficiency was a poor prognostic factor for COVID-19. Severe vitamin D deficiency was remarkably evident among the elderly [24]. 

A systematic review and meta-analysis of 14 studies from an observational prospective and retrospective investigation with 999,179 participants indicated that low serum 25(OH)D was associated with higher susceptibility for COVID-19 infection and more severe disease and mortality [25]. Ongoing clinical trials for assessing the role of vitamin D supplementation in treating COVID-19 infections are under way, and the results so far have shown potential for using vitamin D supplementation, particularly for intensive care patients [26].

Mounting evidence from retrospective studies conducted in the United States and Europe indicates that lower vitamin D levels are commonly associated with risk of acquiring, and dying from, COVID-19 among hospitalized patients. Low levels may have some role in determining severity and outcome of COVID-19 [27]. Moreover, vitamin D deficiency is highly prevalent among critically ill patients and could aggravate the clinical outcome of those vulnerable people by increasing infection rates and mortality [28,29]. Supplementation with vitamin D for those susceptible people plays a pivotal role in helping them recover through supporting the immune system [30,31]. Despite the global rollout of vaccinations, the focus is still on additional promising preventive measures, such as using vitamin D to manage COVID-19 [32]. Vitamin D deficiency is a major public health burden in the Middle East, including the United Arab Emirates (UAE), despite abundant year-round sunlight [33,34,35].

Our objective was to assess the association of vitamin D status with COVID-19 disease severity and mortality in a sample of SARS-CoV-2-positive people from the UAE population. The multiethnic differences among the UAE population together with the unique pattern of COVID-19 mortality and severity in the country merit further investigation.

We used *Our World in Data* (Stats. WHO 2021), an online interactive dashboard hosted by Johns Hopkins University, to track reported COVID-19 cases in real time (https://coronavirus.jhu.edu/map.html accessed on 26 April 2021).

## 2. Materials and Methods

### 2.1. Participants and Collecting Samples

**Figure 1:**
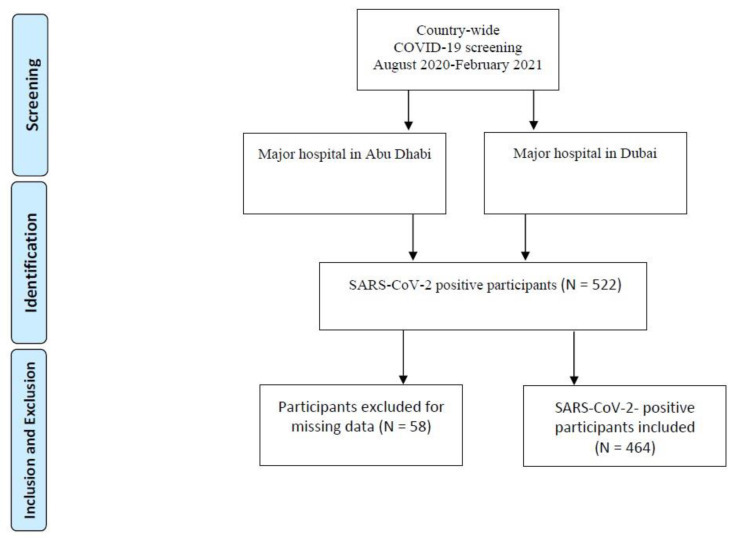
Flowchart diagram for selection of participants.

This study was a multicenter observational study with data collected between August 2020 and February 2021. We recruited 522 participants in the UAE who tested positive for SARS-CoV-2 during a COVID-19 screening at either Sheikh Khalifa Medical Centre in Abu Dhabi, or Rashed hospital in Dubai. We obtained written informed consent from all participants. Inclusion criteria were UAE residency and an age of 18 years or older. Blood samples and nasal swabs were collected for examination. Of 522 participants, 58 had missing data for body mass index (BMI; kilograms per square meter of body surface area), so only 464 were included for data analysis. A flow chart for the selection of participants is shown in Figure 1. This study was approved by the Abu Dhabi Health COVID-19 Research Ethics Committee (DOH/DQD/2020/538), the Dubai Scientific Research Ethics Committee (DSREC-04/2020_09), and the SEHA Research Ethics Committee (SEHA-IRB-005).

### 2.2. Collecting Demographic Data

Demographic, clinical, and outcome data of COVID-19 patients were gathered from questionnaires administered by medical staff at the hospital. Health care providers assessed patients for discharge, including determining disease severity (mild, moderate, or severe). Smoking status was coded as current smoker or nonsmoker. Chest X-ray and/or computed tomography scans were performed in all COVID-19 patients. Concerning immunomodulatory therapy, patients were selected for tocilizumab according to our institutional protocol. The endpoint variable for COVID-19 severity was defined as admission to the intensive care unit, requirement of mechanical ventilation, or death.

### 2.3. Extracting and Quantifying SARS-CoV-2 Viral RNA

An experienced phlebotomy nurse collected blood. A total of 2 mL of blood was collected from the cubital vein by using a gold-top (serum separator) tube. Samples were stored in a sealed biohazard bag and transported at 4 °C in a cool transport container to the Khalifa University Center for Biotechnology’s laboratory for a second confirmatory testing for SARS-CoV-2. Viral RNA was extracted from swab by using the Miracle-AutoXT Automated Nucleic Acid Extraction System (iNtRON Biotechnology, Seoul, South Korea). Genesig from the Primerdesign reverse transcription-PCR COVID-19 detection kit (Watchmoor Point, UK) was used to quantify the viral RNA. PCRs were performed according to the manufacturer’s instructions. Quantitative reverse transcription-PCR was performed using the Magnetic Induction Cycler PCR Machine (MiC) (Bio Molecular Systems, Queensland, Australia).

### 2.4. Measuring Serum 25(OH)D Levels

To assess vitamin D status, we measured the levels of total 25(OH)D. At recruitment, serum samples (that were collected from participants immediately upon arrival to testing centers) were cryopreserved at –80 °C in gel tubes and were used to measure 25(OH)D concentrations with automated electrochemiluminescence (Elecsys 2010; Roche Diagnostics, GmbH, Mannheim, Germany). The detection limit of serum 25(OH)D was 4 ng/mL. The intra-assay coefficient of variation was 5%, and the interassay coefficient was 7.5%.

### 2.5. Defining Severity of Infection

Clinical assessments of participants included determining the severity (mild, moderate, or severe) and diagnosis of pneumonia, confirmed using a chest X-ray. Although participants who presented with mild or no symptoms did not require hospitalization, they were included in the study because they tested positive for SARS-CoV-2. The moderate group had symptoms such as fever, cough, and pneumonia, requiring hospitalization. The severe group presented with critical clinical features, such as high temperature, cough, pneumonia, and shortness of breath, requiring intensive care (World Health Organization, 2020).

### 2.6. Statistical Analysis

Data were analyzed using SPSS version 27.0 (IBM, Armonk, NY, USA). Categorical variables were presented as frequencies and percentages. Continuous variables were presented as mean ± standard deviation. Patients were grouped into three categories of serum 25(OH)D levels: <12 ng/mL, 12–20 ng/mL, and ≥20 ng/mL. Differences according to SARS-CoV-2 severity of infection (asymptomatic, mild, moderate, high) were compared using the chi-square test for categorical variables and one-way analysis of variance for continuous variables. Moreover, differences based on mortality (deceased, alive) were explored using the chi-square test or Fisher’s exact test for categorical variables and independent-samples *t*-test for continuous variables. Simple and multivariate ordered logistic regression models were constructed to determine predictors of infection severity. Simple and multivariate binary logistic regression models were considered to identify variables associated with mortality. Associations between risk factors and outcomes were presented as ORs and 95% CIs, with *p* < 0.05 considered statistically significant.

## 3. Results

We included 464 participants for data analysis and excluded 58 because of missing BMI data. Table 1 summarizes the main demographic and clinical characteristics of participants according to SARS-CoV-2 severity of infection and mortality. The mean age was 47 ± 15 years, with more than 60% of patients being male. Significant differences were observed for age, nationality, chronic disease (type 2 diabetes mellitus (T2D), cardiac disease, and renal disease), smoking, and BMI. The main comorbidities, T2D (32.8%), cardiac disease (11.6%), and renal disease (8.8%), were more prevalent among patients in the severe category. Similarly, those patients were older and more obese than others who had either asymptomatic or mild or moderate COVID-19. About 59% of patients who had vitamin D deficiency and severe vitamin D deficiency had severe symptoms of COVID-19 infection.

A total of 155 (33.4%) patients were vitamin D sufficient, whereas others were either deficient or severely deficient. In total, 65 (14%) UAE nationals were included in the study, of whom 25 (38.5%) had severe infection. The number of Southeast Asian patients was 276 and accounted for 59.5% of all patients, with 72 (26.1%) severely affected by COVID-19. In addition, patients who had severe infection were older and more obese than others (*p* < 0.001). We also evaluated differences in demographics and clinical investigations for patients according to mortality (Table 1). The baseline features differed significantly only in terms of age and major comorbidities. A total of 26 (5.6%) of 464 subjects died.

In Table 2, predictors for severity of infection were determined using multivariate ordered logistic regression analysis with both the adjusted and unadjusted models. To adjust for confounding factors, we used two models: model 1 adjusted for age, sex, and smoking, whereas model 2 adjusted for age, sex, smoking, and comorbidities. BMI > 30 kg/m^2^ (obesity) was significant in the unadjusted model (OR = 2.42 (95% CI, 1.68, 3.49); *p* < 0.001). That factor was not included in the adjusted model. Patients’ sex was not a significant risk factor, whereas smoking and comorbidities lost significance of effect upon adjustment. By contrast, age stood out as a strong independent predictor in both models 1 (OR = 1.08 (95% CI, 1.07, 1.10); *p* < 0.001) and 2 (OR = 1.07 (95% CI, 1.06, 1.09); *p* < 0.001). Serum 25(OH)D levels of < 12 ng/mL in model 1 (OR = 1.79 (95% CI, 1.21, 2.64); *p* = 0.003) and model 2 (OR = 1.76 (95% CI, 1.19, 2.61); *p* = 0.005) were strongly associated with severity of COVID-19.

Predictors for mortality, obtained using binary regression analysis with the outcomes of deceased or alive, are shown in Table 3. Age was strongly associated with risk of mortality. The only other significant predictor in the adjusted model was serum 25(OH)D levels < 12 ng/mL, which were associated with 2.55 times higher risk for death upon adjustment for age and sex (OR = 2.55 (95% CI, 1.03, 6.33); *p* = 0.04) and 2.58 times higher risk for death upon adjustment for age, sex, and comorbidities (OR = 2.58 (95% CI, 1.01, 6.62); *p* = 0.048). Major comorbidities were risk factors in the unadjusted models only. No deceased case patients were smokers; hence, smoking as a risk factor for mortality was not applicable. BMI > 30 kg/m^2^ was not correlated with risk of death in the unadjusted model. For model 2, obesity was utilized in the unadjusted model only and excluded from the adjusted because there is a strong inverse correlation between 25(OH)D and BMI, and confounding factors that affect the factor of interest should not be included for adjustment [36,37].

## 4. Discussion

Several recent reviews highlighted the important role of micronutrients in supporting the immune system and, hence, potentially reducing the risk of COVID-19 infection. Among those, vitamin D is the most attractive for research [38,39,40].

In our study, serum 25(OH)D levels were associated with severity of COVID-19 infection after adjustment for the main confounding factors, namely, age and sex. The protective effect for vitamin D supplementation against viral respiratory infections has been well established, and similar results have started to emerge for COVID-19 [7]. Age was strongly associated with severity and mortality. Our results are in accordance with other observational studies indicating that vitamin D deficiency is significantly associated with COVID-19 severity and death [7]. Age, obesity, and vitamin D deficiency have been well-established risk factors for COVID-19 infection [41]. Aging, also known as senescence, is a complex phenomenon involving many changes in all physiological systems [42,43,44]. The immune system is one system that exhibits several changes during the life span [45,46,47,48]. Being an intricate system that protects the body from external and internal invaders makes it of particular interest to study in the context of aging. Immunosenescence is a term that encompasses the major changes that happen to the immune system during aging, which is characterized by a drop in various immune variables. Recent studies suggest that the most featured changes that happen during aging in the adaptive immune system define the state of immunosenescence [49,50]. One of the most important perceptions in recent years is that the innate immune system has a type of memory, called trained innate immune memory, which at least partially illustrates some of the immune-related features of aging [51,52]. The suggestion of trained innate memory may clarify why aging innate immune cells stay activated [53]. Another study suggests that this state of activation is maintained even in the absence of a specific challenge [47]. The chronic low-grade inflammation (inflamm-aging) is responsible for maintaining immune cells in the activation state. In addition, anti-inflammatory molecules are needed during aging to balance that state, the destruction of which may destroy the whole creature [54].

The association between vitamin D status at time of hospitalization and sequels of acute inflammatory illness could be bidirectional. Even though inflammation could lower the level of serum 25(OH), the immunomodulatory effects of vitamin D are probably the results of its long-term rather than short-term actions [55]. Serum 25(OH)D concentrations decrease near the onset of acute inflammatory illnesses. However, the effect appears short-lived, perhaps only for a few hours [56]. Obesity is another notable factor that has been profoundly associated with COVID-19 risk [57,58,59,60,61,62]. However, recent studies indicate that BMI should be used in the models with confounders to interpret COVID-19 outcomes [57].

The UAE has abundant sunlight throughout most of the year, yet the population is mostly deficient owing to several risk factors, including style of dress and avoidance of sun exposure [33,34]. A retrospective study of 60,979 people from the UAE reported the mean value for serum 25(OH)D to be 48.89 nmol/L. Overall, 82% of those examined presented with hypovitaminosis, of whom 26% of females and 18% of males had severe deficiency. That research showed the serious magnitude of this public health burden among the UAE population [34]. The use of vitamin D supplementation with different attitudes toward medical screening and sun exposure upon the incidental identification of vitamin D deficiency during treatment for other conditions among middle-aged and older adults prompted a robust recommendation for supplementation [61]. UAE health care professionals regularly prescribe supplementation for patients with chronic illnesses, including T2D, cardiovascular diseases, and hypertension. Recently, UAE health insurers have excluded vitamin D tests from coverage among the annual health screen for apparently healthy people, resulting in a resurgence of vitamin D deficiency among young adults in comparison with older adults [61]. Our results are in accordance with previous data reported by Haq and colleagues [34]. Their results showed lower mean serum 25(OH)D for the 33–44 age group than for participants 45 and older—most likely because that subpopulation uses supplementation prescribed by health professionals during medical consultation visits.

Our findings reveal strong implications for vitamin D supplementation not only as a preventive strategy against COVID-19 infection, but also to boost immunity during infection. Accumulating positive results about vitamin D supplementation from several RCTs and intervention-based studies prove that supplementation goes beyond simply addressing vitamin D deficiency to being a protective and maybe even therapeutic measure [26]. Many observational studies have reported the strong link between vitamin D status and risk of disease severity among COVID-19 patients. A meta-analysis of 27 studies reported that vitamin D deficiency in patients with COVID-19 was significantly associated with higher risks of severe infection (OR = 1.64; 95% CI, 1.30, 2.09), hospitalization (OR = 1.81; 95% CI, 1.42, 2.21), and mortality (OR = 1.92; 95% CI, 1.06, 2.58) [63]. Many studies worldwide have investigated the same research question but reached inconsistent and non-decisive results, possibly due to different patient characteristics and research designs. A retrospective observational study to determine the positivity rate for SARS-CoV-2 among more than 190,000 patients in the United States estimated seroprevalence to be 9.3% among the population and revealed a significant inverse association with serum 25(OH)D levels independent of latitude, ethnicity, age, and sex [64]. One plausible explanation for the putative protective role of vitamin D and adequate serum 25(OH) against COVID-19 was linked to the compound nitric oxide (NO), which is an important component of the body’s antiviral defense mechanism [65]. NO inhibits replication of SARS-CoV-2 [66] and inactivates or modifies viral replicating proteins [67]. Calcitriol is a direct transcriptional regulator of endothelial NO synthase, the primary source of NO in the blood. NO reduces risk of arterial stiffness, an important risk factor for hypertension [68]. Hypertension is an important risk factor for COVID-19, and UV exposure can reduce blood pressure [69]. Similar research from the Middle East region and Gulf countries is limited. A comprehensive study in Israel among 14,000 participants showed that vitamin D deficiency was a strong risk factor for COVID-19 infection [70]. Another study among 73 seropositive Iranian patients showed that vitamin D deficiency correlated with mortality [71]. A recent investigation in Saudi Arabia showed a robust association for severe vitamin D deficiency with death but not severity of disease [72]. In a different case–control study in the same country, 138 mildly affected patients were matched with 82 negative controls, and serum 25(OH)D levels were significantly lower in affected people but were not a predictor of disease outcome. That finding called for additional large population-based RCTs to further confirm the results [73].

## 5. Strengths and Limitations

To our knowledge, this is the first study to evaluate 25(OH)D levels in patients who tested positive for SARS-CoV-2 and to examine their association with COVID-19 severity and mortality among a sample of affected people within the UAE. Our findings offer promising results that warrant further research to examine whether vitamin D supplementation could help reduce COVID-19 severity and risk of infection in this population. Vitamin D deficiency is often associated with several comorbidities such as cardiometabolic disorders, T2D, and obesity. The use of multivariate analysis to control for confounding variables and the fact that we recruited subjects from two main hospitals in the UAE’s two main cities (Abu Dhabi and Dubai) strengthened our investigation. Moreover, all nationalities were included to reflect the multiethnic UAE population.

Some limitations are worth noting, however. The small number of deaths in our study most likely affected the analysis. Examining a larger sample to include more mortalities could offer more conclusive results about the relation between vitamin D status and the death outcome from COVID-19 infection in the UAE. The socioeconomic status for all participants was not assessed, but could have affected the dietary habits and availability of fortified foods, which in turn could have affected vitamin D status along with any use of supplements and sun exposure that were not recorded. In addition, the optimal concentration of serum 25(OH)D for overall health remains controversial and using different cutoffs might slightly change results. The bone-centric guidelines recommend a target 25(OH)D concentration of 20 ng/mL (50 nmol/L) and age-dependent daily vitamin D doses of 400–800 IU. The guidelines focused on the pleiotropic effects of vitamin D recommend a target 25(OH)D concentration of 30 ng/mL (75 nmol/L) and age-, body weight-, disease status-, and ethnicity-dependent vitamin D doses between 400 and 2000 IU/day [74]. However, mounting evidence indicates that optimal 25(OH)D levels are 40–60 ng/mL, as seen in the SARS-CoV-2 seropositivity study by Kaufman and colleagues [64], an open-label vitamin D supplementation-breast cancer incidence study [75], and an open-label vitamin D supplementation-blood pressure study [76].

## 6. Conclusions

Our data showed that serum 25(OH)D levels <12 ng/mL are strongly associated with COVID-19 severity and mortality among a sample of affected people in the UAE. Such findings suggest important implications that vitamin D supplementation could help reduce the severity of COVID-19 disease and risk of infection. Further larger observational studies and RCTs are needed to furnish a comprehensive picture about the link between vitamin D and COVID-19 severity and death among the UAE population.

## Figures and Tables

**Table 1:** Characteristics of COVID-19 patients according to disease severity and mortality.

		Severity					Mortality		
	*N*	Asymptomatic	Mild	Moderate	High	*p*	Alive	Deceased	*p*
Total	464	91 (19.6)	99 (21.3)	129 (27.8)	145 (31.3)		438 (94.4)	26 (5.6)	
Age (years)	46.6 ± 14.9	34.3 ± 7.2	41.6 ± 13.7	49.0 ± 13.0	55.7 ± 14.1	**<0.001**	45.7 ± 14.5	62.5 ± 13.1	**<0.001**
Sex									
Female	92 (19.8)	27 (29.3)	20 (21.7)	14 (15.2)	31 (33.7)	**0.006**	89 (96.7)	3 (3.3)	0.28
Male	372 (80.2)	64 (17.2)	79 (21.2)	115 (30.9)	114 (30.6)		349 (93.8)	23 (6.2)	
BMI (kg/m^2^)	28.1 ± 5.9	25.6 ± 4.0	27.0 ± 5.4	28.3 ± 5.4	30.2 ± 6.8	**<0.001**	28.0 ± 5.7	30.2 ± 8.5	0.07
Obesity (BMI > 30 kg/m^2^)									
Obese	136 (29.3)	11 (8.1)	23 (16.9)	44 (32.4)	58 (42.6)	**<0.001**	128 (94.1)	8 (5.9)	0.89
Not obese	328 (70.7)	80 (24.4)	76 (23.2)	85 (25.9)	87 (26.5)		310 (94.5)	18 (5.5)	
Nationality									
UAE	65 (14)	0 (0)	13 (20)	27 (41.5)	25 (38.5)	**<0.001**	59 (90.8)	6 (9.2)	0.25
Arab (Middle Eastern)	103 (22.2)	8 (7.8)	41 (39.8)	17 (16.5)	37 (35.9)		97 (94.2)	6 (5.8)	
Asian	276 (59.5)	81 (29.3)	44 (15.9)	79 (28.6)	72 (26.1)		264 (95.7)	12 (4.3)	
Others	20 (4.3)	2 (10)	1 (5)	6 (30)	11 (55)		18 (90)	2 (10)	
Current Smoker									
Yes	50 (10.8)	13 (26)	23 (46)	11 (22)	3 (6)	**<0.001**	50 (100)	0 (0)	0.10
No	414 (89.2)	78 (18.8)	76 (18.4)	118 (28.5)	142 (34.3)		388 (93.7)	26 (6.3)	
25(OH)D Level (ng/mL)									
<12	127 (27.4)	21 (16.5)	28 (22)	34 (26.8)	44 (34.6)	0.07	117 (92.1)	10 (7.9)	0.32
12–20	182 (39.2)	48 (26.4)	36 (19.8)	53 (29.1)	45 (24.7)		175 (96.2)	7 (3.8)	
≥20	155 (33.4)	22 (14.2)	35 (22.6)	42 (27.1)	56 (36.1)		146 (94.2)	9 (5.8)	
Cardiac Disease									
Yes	54 (11.6)	0 (0)	10 (18.5)	16 (29.6)	28 (51.9)	**<0.001**	44 (81.5)	10 (18.5)	**<0.001**
No	410 (88.4)	91 (22.2)	89 (21.7)	113 (27.6)	117 (28.5)		394 (96.1)	16 (3.9)	
Chronic Lung Disease									
Yes	24 (5.2)	0 (0)	2 (8.3)	8 (33.3)	14 (58.3)	**0.004**	20 (83.3)	4 (16.7)	**0.04**
No	439 (94.8)	91 (20.7)	97 (22.1)	120 (27.3)	131 (29.8)		417 (95)	22 (5)	
Diabetes									
Yes	152 (32.8)	4 (2.6)	23 (15.1)	56 (36.8)	69 (45.4)	**<0.001**	136 (89.5)	16 (10.5)	**0.001**
No	312 (67.2)	87 (27.9)	76 (24.4)	73 (23.4)	76 (24.4)		302 (96.8)	10 (3.2)	
Renal Disease									
Yes	41 (8.8)	0 (0)	3 (7.3)	12 (29.3)	26 (63.4)	**<0.001**	33 (80.5)	8 (19.5)	**<0.001**
No	423 (91.2)	91 (21.5)	96 (22.7)	117 (27.7)	119 (28.1)		405 (95.7)	18 (4.3)	
Metabolic Disease									
Yes	27 (5.8)	1 (3.7)	4 (14.8)	8 (29.6)	14 (51.9)	**0.04**	23 (85.2)	4 (14.8)	0.06
No	437 (94.2)	90 (20.6)	95 (21.7)	121 (27.7)	131 (30)		415 (95)	22 (5)	
Liver Disease									
Yes	7 (1.5)	0 (0)	1 (14.3)	2 (28.6)	4 (57.1)	0.41	6 (85.7)	1 (14.3)	0.33
No	457 (98.5)	91 (19.9)	98 (21.4)	127 (27.8)	141 (30.9)		432 (94.5)	25 (5.5)	

Data are presented as *N* (%); data presented as mean ± SD; BMI, body mass index; UAE, United Arab Emirates; 25(OH)D, serum 25-hydroxyvitamin D; *p* < 0.05 considered significant (shown in boldface).

**Table 2:** Predictors for COVID-19 severity using multivariate ordered logistic regression analysis.

			Model 1		Model 2	
	Unadjusted		Adjusted		Adjusted	
Predictor	OR (95% CI)	*p*	OR (95% CI)	*p*	OR (95% CI)	*p*
Age	1.08 (1.07, 1.10)	**<0.001**	1.08 (1.07, 1.10)	**<0.001**	1.07 (1.06, 1.09)	**<0.001**
Male	1.39 (0.91, 2.14)	0.13	1.22 (0.78, 1.91)	0.38	1.23 (0.78, 1.94)	0.38
Smoker	0.34 (0.21, 0.57)	**<0.001**	0.60 (0.35, 1.02)	0.06	0.60 (0.35, 1.02)	0.06
Obese (BMI > 30 kg/m^2^)	2.42 (1.68, 3.49)	**<0.001**				
Cardiac Disease	3.11 (1.84, 5.26)	**<0.001**			0.72 (0.38, 1.37)	0.32
Chronic Lung Disease	3.96 (1.81, 8.67)	**0.001**			1.64 (0.68, 3.93)	0.27
Diabetes	3.68 (2.56, 5.29)	**<0.001**			1.27 (0.82, 1.97)	0.28
Renal Disease	5.13 (2.71, 9.73)	**<0.001**			1.66 (0.80, 3.48)	0.18
Metabolic Disease	2.79 (1.35, 5.75)	**0.005**			1.34 (0.60, 2.99)	0.45
Liver Disease	3.35 (0.81, 13.85)	0.10			2.99 (0.54, 16.52)	0.21
25(OH)D < 12 ng/mL	1.22 (0.84, 1.76)	0.29	1.79 (1.21, 2.64)	**0.003**	1.76 (1.19, 2.61)	**0.005**
25(OH)D < 20 ng/mL	0.71 (0.50, 1.00)	0.051	1.17 (0.80, 1.71)	0.41	1.14 (0.78, 1.66)	0.51

Model 1 is adjusted for age, sex, and smoking status. Model 2 is adjusted for age, sex, smoking status, and comorbidities. Data are presented as frequencies (%) and odds ratio (OR) (95% CI); BMI, body mass index; 25(OH)D, serum 25-hydroxyvitamin D; *p* < 0.05 considered significant (shown in boldface).

**Table 3:** Significant predictors of mortality, using binary logistic regression analysis.

			Model (1)		Model (2)	
	Unadjusted		Adjusted		Adjusted	
Predictor	OR (95% CI)	*p*	OR (95% CI)	*p*	OR (95% CI)	*p*
Age	1.08 (1.05, 1.11)	**<0.001**	1.08 (1.05, 1.12)	**0.001**	1.07 (1.03, 1.11)	**0.001**
Male	1.96 (0.57, 6.66)	0.28	1.67 (0.46, 6.02)	0.43	1.84 (0.47, 7.25)	0.38
Smoker	NA					
Obese (BMI > 30 kg/m^2^)	1.08 (0.46, 2.54)	0.87				
Cardiac Disease	5.60 (2.39, 13.08)	**<0.001**			1.66 (0.57, 4.83)	0.35
Chronic Lung Disease	3.79 (1.19, 12.04)	0.02			1.12 (0.28, 4.41)	0.87
Diabetes	3.55 (1.57, 8.03)	**0.002**			0.99 (0.38, 2.58)	0.98
Renal Disease	5.45 (2.21, 13.49)	**<0.001**			1.33 (0.45, 3.95)	0.60
Metabolic Disease	3.28 (1.04, 10.31)	**0.04**			2.45 (0.64, 9.34)	0.19
Liver Disease	2.88 (0.33, 24.85)	0.34			1.66 (0.16, 17.41)	0.67
25(OH)D < 12 ng/mL	1.71 (0.76, 3.89)	0.20	2.55 (1.03, 6.33)	**0.04**	2.58 (1.01, 6.62)	**0.048**
25(OH)D < 20 ng/mL	0.94 (0.41, 2.17)	0.89	1.72 (0.68, 4.34)	0.25	1.71 (0.66, 4.43)	0.27

Model 1 is adjusted for age and sex. Model 2 is adjusted for age, sex, and comorbidities. Data are presented as frequencies (%) and odds ratio (OR) (95% CI); BMI, body mass index; 25(OH)D, serum 25-hydroxyvitamin D; *p* < 0.05 considered significant (shown in boldface).

## Data Availability

Data can be available upon request from the first and corresponding authors.
